# Effects of early antiplatelet therapy after splenectomy with gastro‐oesophageal devascularization

**DOI:** 10.1111/ans.14395

**Published:** 2018-02-03

**Authors:** Jin‐Bao Zhou, Bao‐Yang Luo, Chi‐Wen Liu, Feng Zhu

**Affiliations:** ^1^ Department of General Surgery The Third Affiliated Hospital of Soochow University Changzhou China; ^2^ Department of General Surgery Sir Run Run Hospital of Nanjing Medical University Nanjing China

**Keywords:** antiplatelet therapy regimen, portal hypertension, portal vein hypertension, risk factors, splenectomy with gastro‐oesophageal devascularization

## Abstract

**Background:**

This study aimed to explore the effects of early antiplatelet therapy (APT) for portal vein thrombosis (PVT) in patients with cirrhotic portal hypertension after splenectomy with gastro‐oesophageal devascularization.

**Methods:**

We retrospectively analysed 139 patients who underwent splenectomy with gastro‐oesophageal devascularization for portal hypertension due to cirrhosis between April 2010 and December 2016. Based on the post‐operative platelet values, we used two different APT regimens: APT was started when platelet counts were increased to 200 × 10^9^/L or above (group A, *n* = 64) or 300 × 10^9^/L or above (group B, *n* = 75). We took note of the patients’ clinical symptoms, operative factors and biochemical indicators.

**Results:**

Platelet count, mean platelet volume, D‐dimer and pancreatic fistula were closely related to the development of PVT. Early APT was an independent protective factor for PVT. The incidence of post‐operative PVT was 15.1% (21/139) overall, 4.7% (3/64) in group A and 24% (18/75) in group B; there was a significant difference between groups A and B (*χ* ^2^ = 10.042, *P* = 0.002).

**Conclusion:**

Platelet count, mean platelet volume, D‐dimer and pancreatic fistula were independent risk factors for the development of PVT after splenectomy with gastro‐oesophageal devascularization. Selection of the appropriate timing for early APT according to the post‐operative platelet count was feasible. Moreover, the use of aspirin combined with dipyridamole was safe and effective for early prevention of PVT.

## Introduction

Although transjugular intrahepatic portosystemic shunt and liver transplantation have been gradually applied for the treatment of portal hypertension, splenectomy with gastro‐oesophageal devascularization remains the most important modality that can prevent and control the risk for infection and gastrointestinal bleeding, relieve symptoms of hypersplenism, moderate portal vein pressure and improve patients’ quality of life.^1^ However, the development of portal vein thrombosis (PVT) is often difficult to avoid after splenectomy with gastro‐oesophageal devascularization. If not treated appropriately, PVT is potentially fatal by further increasing portal vein pressure and deteriorating liver function, which may increase the risk for upper gastrointestinal bleeding, hepatic coma or even fatal intestinal necrosis.^2^ In fact, the natural incidence rate of PVT in patients with liver cirrhosis was reported to be 6.6%, which can increase to 18.9% to 57% after splenectomy.^3^ Therefore, it is important to analyse the related risk factors and preventive measures of PVT after splenectomy with gastro‐oesophageal devascularization.

The early symptoms of PVT are mild and may be easily overlooked, and once the symptoms of serious complications set in, the treatment ideal interventions are rarely available. At present, there are no commonly accepted standards to evaluate the risk factors and prevention strategies for PVT,^4^ although prophylactic anticoagulation therapy remains the primary method to prevent PVT after splenectomy with gastro‐oesophageal devascularization. The most frequently used drugs are low‐molecular weight heparin, the vitamin K antagonist warfarin and aspirin.^5^ However, the use of these drugs is not standard. Therefore, we aimed to retrospectively study the efficacy of early antiplatelet therapy (APT) for PVT in 139 patients with cirrhosis who underwent splenectomy with gastro‐oesophageal devascularization.

## Methods

### Inclusion and exclusion criteria

This study included all patients who had varying degrees of hypersplenism and oesophageal varices before surgery. No patient had PVT before surgery, as revealed by colour Doppler ultrasound or abdominal computed tomography angiography. The exclusion criteria were the presence of liver cirrhosis complicated with liver cancer or other malignant tumours; blood clot before surgery and preoperative systemic vital organ dysfunction; liver cirrhosis associated with abdominal trauma such as splenic and hepatic rupture that required splenectomy to prevent haemorrhagic shock; severe underlying diseases such as cardiovascular diseases and respiratory diseases; and incomplete clinical data. Finally, 139 patients were included in our study.

### Data collection

The following data were recorded: gender, age, aetiology, concomitant diabetes, hypertension, prothrombin time, ascites, pancreatic fistula, D‐dimer, history of upper gastrointestinal bleeding, degree of oesophageal and gastric varices, grade of preoperative hepatic functional reserve, surgical approach, operative time, blood loss, post‐operative anticoagulation and haemostasis. In the first week after surgery, platelet count, mean platelet volume (MPV), platelet distribution width and D‐dimer were measured and collected every other day. Platelet count was also measured once a month in the first year after surgery. The higher value was used if more than two sets of test results were obtained.

### Treatment and grouping

In our study, the platelet count and coagulation function of all patients were monitored every other day in the first week, and then monthly in the first year after surgery. The patients were classified into two groups according to the timing of prophylactic APT. Group A comprised patients who were given early prophylactic APT with aspirin 100 mg daily for 1 year when the post‐operative platelet count increased to 200 × 10^9^/L or above; dipyridamole 50 mg daily was added if the post‐operative platelet count continued to increase rapidly. Group B comprised patients who received advanced APT with the same regimen when the post‐operative platelet count increased to 300 × 10^9^/L or above. The doses of aspirin and dipyridamole were adjusted as necessary according to the prothrombin time and international normalized ratio (PT‐INR) and platelet count. In patients who were at risk for bleeding, dipyridamole was reduced to 25 mg daily or discontinued. In patients with persistently increasing platelet count, hydroxyurea 50 mg twice a day was temporarily added. If no haemorrhagic tendency was identified, the APT regimen was continued for 1 year; thereafter, aspirin and dipyridamole were discontinued if the patients’ platelet count normalized. In patients with persistently increased platelet count, aspirin and dipyridamole were given on a long‐term basis.

Colour Doppler ultrasound was repeated monthly in the first year. Once PVT was confirmed, the patients were administered early thrombolytic therapy with urokinase via the peripheral venous route at a bolus dose of 400 000 U within 30 min, followed by continuous microinfusion of 30 000–50 000 U per hour for 3–5 days. Following thrombolytic treatment, the patients were administered oral warfarin 3.0 mg one to two times daily and aspirin 100 mg daily for anticoagulation therapy. When the serial Doppler ultrasound examinations showed complete or partial dissolution of the target thrombus, the treatment was considered effective and was switched to oral warfarin monotherapy 3.0 mg one to two times daily for 1 month; in cases that showed little change or even enlargement of the target thrombus, thrombolytic therapy was defined as ineffective and the patients were continued on oral warfarin and aspirin and followed up regularly. After discharge, we continued to perform B‐mode ultrasound every month until the end of the follow‐up period of 6 months to 2 years.

### Statistical analysis

The Statistical Package for the Social Sciences (SPSS) version 20 software (SPSS Inc., Chicago, IL, USA) was used for statistical analyses. The measured data were expressed as mean ± standard deviation. Count data were presented as the number of cases and percentage (%). The Student's *t*‐test and chi‐square test were performed to analyse the differences between the PVT and non‐PVT groups. Multivariate logistic regression analysis was conducted to analyse the independent factors for PVT. Logistic regression prediction model was established and a logit *P*‐value was calculated. *P* < 0.05 was considered statistically significant.

## Results

### General information on the subjects

The study included a total of 139 cirrhotic patients, including 72 men and 67 women, with a median age of 55 years (range: 22–76 years); 129 of these cirrhosis cases were hepatitis B virus‐related and 10 were hepatitis C virus‐related. The liver function grade was Child–Pugh A in 65 patients and Child–Pugh B in 74 patients. The approach to splenectomy with gastro‐oesophageal devascularization was open surgery in 93 patients (66.9%) and laparoscopic in 46 patients (33.1%). All major clinical parameters were not significantly different among the four groups.

### Incidence of PVT after splenectomy with gastro‐oesophageal devascularization

According to the occurrence of PVT, the patients were divided into the PVT group (*n* = 21) and the non‐PVT group (*n* = 118). The incidence of post‐operative PVT was 15.1% (21/139) in all patients and was significantly lower in group A than in group B (4.7% (3/64) versus 24% (18/75); *χ* ^2^ = 10.042, *P* = 0.002). Multivariate logistic regression analysis showed that early APT was an independent positive predictor of PVT (*P* = 0.022). The results suggested the efficacy of our method of early APT, particularly in group A patients in whom the incidence of PVT was significantly reduced.

### Relationship between the clinical features and the occurrence of PVT

Comparison of the clinical features of patients according to the development of PVT (Table 1) showed that the factors significantly related to the development of PVT were operation time, splenic weight, pancreatic fistula, platelet count, MPV, D‐dimer, platelet distribution width and APT between the PVT and non‐PVT groups (*P* < 0.05).

**Table 1:** Comparison of the clinical features according to the presence of PVT

Clinical features		PVT group (*n* = 21)	Non‐PVT group (*n* = 118)	*P‐*value
Gender	Male/Female	11/10	61/57	0.572
Age (year)		51.48 ± 10.02	53.50 ± 11.04	0.431
Child–Pugh score	Child–Pugh A	8	57	0.267
	Child–Pugh B	13	61	
Aetiology	HBV/HCV	20/1	109/9	0.535
Operation mode	LS/OS	8/13	38/80	0.384
Operation time (min)		156 ± 59	194 ± 76	0.016
Intraoperative blood loss (mL)		536 ± 166	544 ± 76	0.967
Hypertension	Yes/no	2/19	23/95	0.222
Diabetes	Yes/no	2/19	14/104	0.552
Ascites	Yes/no	12/9	66/52	0.556
Pancreatic fistula	Yes/no	6/15	10/108	0.024
Decompensated cirrhosis	Yes/no	15/6	92/26	0.343
PT (s)		13.18 ± 3.13	13.89 ± 1.78	0.136
D‐dimer (mg/L)		7.54 ± 4.04	5.19 ± 3.91	0.018
PLT (×10^11^/L)		3.45 ± 0.25	2.70 ± 0.13	0.013
Splenic weight (g)		801 ± 363	643 ± 313	0.039
APT	Group A/B	3/18	61/57	0.002
MPV (fL)		12.45 ± 1.73	10.79 ± 1.98	0.001
PDW (%)		19.48 ± 3.03	17.49 ± 2.82	0.004

APT, antiplatelet therapy; HBV, hepatitis B virus; HCV, hepatitis C virus; LS, laparoscopic splenectomy; MPV, mean platelet volume; OS, open splenectomy; PDW, platelet distribution width; PLT, platelet count; PT, prothrombin time; PVT, portal vein thrombosis.

### Analysis of the independent influential factors for PVT

In the multivariate logistic regression analysis (Table 2) of the significant factors in the univariate analysis, platelet count, MPV, D‐dimer and early APT were significantly different between the PVT and non‐PVT groups (*P* < 0.05). Among these factors, pancreatic fistula, platelet, MPV and D‐dimer were the independent risk factors for post‐operative PVT occurrence, with odds ratios of >1. Early APT was an independent protective factor for post‐operative PVT occurrence, with odds ratio of <1. These results indicated that pancreatic fistula, platelet count, MPV, D‐dimer and early APT were the independent factors that influenced the development of PVT.

**Table 2:** Multivariate logistic regression analysis

Variables	Regression coefficient (*β* value)	Standard error	Wald value	Degree of freedom	*P*	Odds ratio	95% confidence interval
PLT	0.007	0.003	6.770	1	0.009	1.007	1.002–1.013
MPV	0.467	0.193	5.816	1	0.016	1.594	1.091–2.330
D‐dimer	0.197	0.075	6.927	1	0.008	1.218	1.052–1.411
PF	2.130	0.876	5.907	1	0.015	8.414	1.510–46.875
APT	−1.857	0.811	5.246	1	0.022	0.156	0.032–0.765

APT, antiplatelet therapy; MPV, mean platelet volume; PF, pancreatic fistula; PLT, platelet count.

### Relationship between complications and PVT

The spleen is the largest immune organ in the body, accounting for 25% of the total lymphoid tissue; therefore, secondary infection is the most important complication after splenectomy with gastro‐oesophageal devascularization. In our study, although several measures were taken to prevent post‐operative infection, seven patients developed abdominal infection; four of them (two in group A and two in group B) subsequently developed PVT. Coincidentally, all four patients had accompanying pancreatic fistula. Ignore the effects of complications such as pancreatic fistula and infection, it can see that patient with PVT in group A was significantly less than group B. Therefore, early APT was clearly effective for prevention of post‐operative PVT. There were 10 patients who had one or a combination of non‐specific clinical symptoms of loss of appetite, abdominal pain and abdominal distension.

### Management and outcome of patients

After splenectomy with gastro‐oesophageal devascularization, the platelet count usually returns to normal from 3 months to 1 year. In our study, the platelet count returned to normal within 1 year in 124 of 139 patients, including 59 (92.18%, 59/64) patients in group A and 63 (84%, 63/75) patients in group B (*χ* ^2^ = 0.374, *P* = 0.541). In addition, during the APT, only a small number of patients needed adjustment of the APT regimen; specifically, there was a need to reduce the amount of dipyridamole or to add hydroxyurea in seven and three patients, respectively, in group A and in nine and four patients, respectively, in group B.

All 21 patients with post‐operative PVT were treated with thrombolytic, anticoagulant and anti‐aggregation agents. Among the 21 patients, complete or partial thrombus dissolution was achieved in 18 patients, including three (100%, 3/3) patients in group A and 15 (83.33%, 15/18) patients in group B (*χ* ^2^ = 0.583, *P* = 0.445). During thrombolytic therapy, epistaxis and subcutaneous ecchymosis occurred in two patients, including one in group A and one in group B. In such cases, thrombolytic therapy was discontinued and haemostatic agents were administered immediately; bleeding was successfully controlled and the patients recovered well. Finally, during the 2 years of follow‐up, the overall mortality rate was 2.1% (3/139); one patient in group A died of cardiopulmonary disease and two patients in group B died of cancer and cardiovascular disease.

## Discussion

Splenomegaly is considered a risk factor for PVT after splenectomy with gastro‐oesophageal devascularization.^6^ PVT has been reported to potentially lead to severe clinical adverse events or poor outcomes.^7^ Regrettably, the mechanism underlying PVT formation remains unclear, and the prophylactic application of anticoagulants is controversial because of the concerns about the risk of bleeding.^8^ Various prevention schemes have been put forward, but none of them have been effective.^7^, ^9^ Therefore, analysis of the risk factors and prophylaxis of PVT after splenectomy with gastro‐oesophageal devascularization was critical.

Several reported cases of pancreatic fistula were caused by pancreatic tail injury during splenectomy,^10^ but pancreatic fistula is often not taken seriously because it can be resolved by drainage. However, pancreatic fistula extends the hospital stay and can increase the complications of internal bleeding, gastric emptying, thrombosis, abdominal infection, pleural effusion and other surgical‐related complications, and bring physical and mental pain to the patient.^10^ In our study, pancreatic fistula was noted in six of the 21 cases in the PVT group and in only 10 of the 118 cases in the non‐PVT group. The results of this study suggested that the incidence of PVT was significantly higher in patients with pancreatic fistula than in those without pancreatic fistula. Moreover, our data implied that the presence of post‐operative pancreatic fistula could significantly increase the risk for infection and PVT after splenectomy with gastro‐oesophageal devascularization. Fortunately, our data showed that early APT may have a better effect if pancreatic fistula and infection could be prevented.

After splenectomy with gastro‐oesophageal devascularization, platelets can increase rapidly in the short term, with a peak at 7–20 days after surgery.^1^ Platelets can secrete and express a large number of substances that are crucial mediators of coagulation, inflammation and thrombosis.^11^ In our study, the patients who developed PVT had a significantly higher platelet count at 7 days after surgery compared with those who did not develop PVT (*P* = 0.013). Our study also found that the MPV was closely related with platelet activity; likewise, another report demonstrated that MPV was positively associated with the levels of thrombopoietin and interleukin‐6, which are cytokines that regulate megakaryocyte ploidy and platelet number.^12^ When platelets with larger volumes are activated, they can release more thrombotic precursor material and increase the possibility of thrombosis. In addition, a high MPV reflects the extent of vascular injury and inflammation. Vascular endothelial cell injury can release thromboxane and endothelin, which activates the extrinsic coagulation system to form thrombosis. In this study, we found that the patients’ MPV almost did not change before and after surgery, the patients with higher MPV may have a higher probability of thrombosis.

An increasing D‐dimer level was reported to indicate the presence of active clotting and fibrinolysis in the body.^13^ D‐dimer test has been widely employed to predict the occurrence and recurrence of venous thromboembolism and to guide the duration of anticoagulation in patients with venous thromboembolism.^14^ According to our research, the levels of D‐dimer after surgery were generally higher in patients with PVT than in those without PVT. Therefore, a rapid increase in D‐dimer after splenectomy could be an important sign of PVT formation.

Multivariate logistic regression analysis showed that early APT was an independent positive predictor of PVT.^15^ Various prevention protocols have been proposed, but the effectiveness of these protocols varied in the duration and dose of the drugs. Therefore, there had been no generally acceptable PVT prophylactic regimen for all patients. An increased count and augmented aggregation competence of platelets after operation were the important factors related to PVT that should be taken into account when selecting antiplatelet drugs. Aspirin has been applied in the prevention and treatment of thrombotic diseases, with satisfactory safety because of its antiplatelet aggregation competence.^16^ Thromboxane A2 (TXA2) is an important factor in the activation of platelets. Aspirin exerts its antiplatelet effect by inhibiting cyclooxygenase and reducing the synthesis of TXA2.^17^ Dipyridamole and aspirin have a synergistic effect in inhibiting the formation of TXA2, but dipyridamole has a stronger antiplatelet aggregation effect, mainly through inhibition of phosphodiesterase activity, increase in adenosine cyclase activity and increase in the level of platelet endocyclic phosphate. In this study, we selected dipyridamole and aspirin as the regular PVT prevention regimen. Low‐molecular weight heparin was not used in this study because some studies have shown that it can increase the risk of surgical site bleeding.^18^

Our results showed that the early use of aspirin followed by long‐term maintenance therapy with dipyridamole and aspirin significantly reduced the incidence of PVT in group A, compared with group B, without causing major bleeding complications. Notably, the development of pancreatic fistula and infection after splenectomy with gastro‐oesophageal devascularization should be prevented because these can render early APT less effective.

Theoretically, early use of antiplatelet drugs to prevent PVT in cirrhotic portal hypertensive patients after surgery would increase the risk of bleeding. However, previous studies in these patients have demonstrated that early APT after splenectomy with gastro‐oesophageal devascularization was safe and effective in preventing PVT.^19^ Based on our study, regular monitoring and maintenance of the PT‐INR level between 1.25 and 1.5 during APT is necessary in order to guarantee safety.

In conclusion, pancreatic fistula, platelet count, MPV and D‐dimer were the independent risk factors for the development of PVT after splenectomy with gastro‐oesophageal devascularization. Patients with these factors should be carefully examined after surgery. Selection of the appropriate timing of early APT according to the post‐operative platelet count was feasible. Moreover, the use of aspirin combined with dipyridamole was safe and effective for early prevention of PVT. In fact, because of the risk of bleeding, we empirically started APT when the post‐operative platelet count was increased to 200 × 10^9^/L or above. A randomized prospective study with a better design and a longer follow‐up period is needed to validate our conclusions.

## Conflicts of interest

None declared.
